# Genetic and genomic diversity of NheABC locus from *Bacillus* strains

**DOI:** 10.1007/s00203-017-1350-9

**Published:** 2017-03-10

**Authors:** Yan Cai, Tingxuan Huang, Yuekang Xu, Guoping Zhou, Ping Zou, Guifeng Zeng, Xiaojin Liu

**Affiliations:** 1 College of Life Science, Anhui Normal University, No.1# Beijing East Road, Wuhu, 241002 Anhui People’s Republic of China; 2 School of Food Science and Technology, Changzhou University, No.1# Gehu Middle Road, Changzhou, 213164 Jiangsu People’s Republic of China; 3 School of Biology and Pharmaceutical Engineering, Wuhan Polytechnic University, Wuhan, 430023 People’s Republic of China

**Keywords:** *Bacillus*, *NheABC*, Genetic diversity, MLST

## Abstract

Non-hemolytic enterotoxin (NHE), a tri-partite, proteinaceous toxin encoded by contiguous *nheA, nheB* and *nheC* genes of *Bacillus cereus sensu lato* (*B. cereus s.l.*), is considered to be associated with the foodborne diarrheic syndrome. However,* B. cereus s.l.* includes a number of closely related strains, and the occurrence of NHE among them, and other members of *Bacillus* is unclear. Consequently, we aimed to determine the distribution and evolution of NHE within *Bacillus* by confirming the presence of the *nheA, B* and *C* sequences and variation within them using published data, and to analyze the genomic and genetic diversity. The phylogenetic tree of NHE proteins (NheA, NheB and NheC) from 81 different *B. cereus s.l*. strains was constructed. And on the genetic determinants of the NHE toxin did not bring any obvious link between the *nheABC* genes sequence of a strain and its virulence in the diarrhoeal pathogenesis. Analysis of the genomic diversity of the *nheA, B* and *C* loci revealed that their upstream regions were more conserved than the downstream sequences. Multilocus sequence typing schemes (MLST) based on seven concatenated housekeeping genes and *nheA, B* and *C* genes of the 75 strains were developed. The neighbor joining phylogenetic tree based on seven housekeeping genes together with *nheA, B* and *C* genes was similiar to published *Bacillus* phylogenetic trees. And on the genetic determinants of the NHE toxin did not bring any obvious link between the *nheABC* genes sequence of a strain and its virulence in the diarrhoeal pathogenesis.The results indicate that *nheA, B* and *C* genes do not affect the diversity of housekeeping genes, and this specific NHE protein does not participate in the diarrheic syndrome.

**Electronic supplementary material:**

The online version of this article (doi:10.1007/s00203-017-1350-9) contains supplementary material, which is available to authorized users.

## Introduction

*Bacillus cereus s. l*. is widely distributed in food, soil and plants (Okinaka and Keim 2016). This Gram-positive, spore-forming bacterium may behave as an opportunistic human pathogen. Long known to be responsible for two forms of food poisoning, characterized by either diarrhea or nausea and vomiting. The diarrhoeal symptom includes the following symptoms, which usually last generally less than 48 h: abdominal pain, profuse watery diarrhea, sometime nausea and vomiting within 8–16 h. Although most cases are generally mild, more serious and even lethal cases have been reported in Europe.

Concerning the diarrhoeal syndrome, no definitive hypothesis that correlates the symptoms to a unique component exists to date. Indeed, several putative enterotoxins have been reported to be potentially responsible, alone or in combination, of the diarrhoeic pathotypes. These include the tripartite enterotoxins as Haemolysin BL (HBL) and Nonhaemolytic enterotoxin (NHE), but also the single-component toxin, Cytotoxin K (CytK) also sometimes named Haemolysin IV (HlyIV). In addition to these three major candidates, other molecules are also cited as potential enterotoxins involved in the diarrhoeic syndrome, such as enterotoxin FM (EntFM) (Boonchai et al. 2008), enterotoxin S (entS), enterotoxin-T (bceT) and pore-forming haemolysins like the Cereolysin O (CerO), Haemolysin II (HemII) and Haemolysin III (HlyIII). Besides, other virulence factors seem to contribute to the *B. cereus* foodborne diseases, such as phospoholipases or the sphingomyelinase (SMase).

The non-hemolytic enterotoxin NHE is encoded by *nheA, nheB* and *nheC* (Kim et al. 2015). NHE is a tripartite pore-forming toxin that requires the combination of three proteins: NheA, NheB and NheC. NHE was first isolated from the supernatant of a *B. cereus s.l*. culture that caused a large food poisoning outbreak in Norway in 1995 (Lund and Granum 1996). NHE proteins are secreted independently and maximal toxic activity on Vero cells requires all the three parts in a molar ratio 10:10:1 of NheA, NheB and NheC, respectively. NheB is the binding component of the enterotoxin complex and an increase in the concentration of NheC results in a decrease in Nhe toxic activity (Lindbäck et al. 2004).

However, the genes coding for these putative enterotoxins are, for the most part, largely distributed among the *B. cereus* group isolates, irrespective of their diarrheic activities (McIntyre et al. 2008; Swiecicka et al. 2006). Moreover, with the exception of the rabbit ileum assay, no animal model can be used to specifically test for the diarrhoeal properties of the strains (or for purified proteins) (Beecher et al. 1995). Only classical assays on animal cell lines are readily available, but they only give information on the generic cytotoxicity of these putative enterotoxins (Jeßberger et al. 2014).

*B. cereus s.l.* includes a number of closely related species, viz. *Bacillus cereus sensu stricto*, *Bacillus mycoides*, *Bacillus pseudomycoides*, *Bacillus thuringiensis*, *Bacillus weihenstephanensis*, *Bacillus anthracis* and *Bacillus cytotoxicus*, and many strains thereof (Lindbäck et al. 2004). The occurrence of NHE within the group, and other members of *Bacillus* is unclear, e.g., we have found NHE in some non- *B. cereus s.l*. members. Analysis methods such as MLST indexes the sequence variation present in a small number (usually seven) of housekeeping gene fragments located around the bacterial genome, is to provide a highly discriminating typing system that can be particularly helpful for the typing of bacterial pathogens (Keith and Martin 2014). And PHYLOViZ software make the data easy to be visualized and export the results in graphic formats (Alexandre et al. 2012). In this study, we were interested in determining the particular strains that produce NHE and to trace the molecular evolution and variation of the *nheABC* genes and the phylogenetic relationship of the various *Bacillus cereus s.l*. strains to others within *Bacillus*. Our approach involved sequence-based typing analysis, interrogation of online databases of allelic profiles and associated epidemiological data collected.

With the aim of assessing the potential implication of NHE in the diarrheic syndrome, the genomic and genetic diversity, as well as the occurrence and the evolutionary ecology of *nheABC* were studied in detail on a collection of *Bacillus cereus* strains.

## Materials and methods

### Bioinformatics and strains information

The presence of *nheABC* genes and their corresponding putative sequences was screened in the NCBI database among the 174 genomes from the *B. cereus* group as available (Aug. 15, 2015). The NheA, B and C protein sequences from *B. cereus* ATCC14579 were applied to find all the homologous proteins among the 174 strains by BLAST program in http://blast.ncbi.nlm.nih.gov/Blast.cgi.

A set of strains (Table 1) coming from food poisonings (FP), food products (F), the environment (E), clinical cases (C) and unknown origin (U) was collected to investigate the presence of *nheABC* genes. Then, a panel of positive strains was selected for MLST analysis (Table 2 and below). This selection was based on the origin, the year and the country of strains and the species to obtain the most diversified panel of *nheABC* positive strains.

**Table 1:** The occurrence of *nheABC* in all the 92 *B. cereus s.l*. group strains whose whole genome sequence available online

Source	*nheABC* ^+^	*nheABC* ^−^	Positive ratio
C	*Ba* A2012, *Ba* Ames, *Ba* A0193, *Ba* Sterne, *Ba* A0389, *Ba* A0174, *Ba* A0442, *Ba* A0465, *Ba* A0488, *Ba* A1055, *Ba* Australia 94, *Ba* CNEVA-9066, *Ba* Kruger B, *Ba* Vollum, *Ba* WNA USA6153, *Ba* ‘Ames Ancestor’, *Bc* 03BB108, *Bc* 03BB102, *Bc* G9842, *Bc* AH1272, *Bc* AH1273, *Bc* R309803, *Bc* AH820, *Bc* B4264, *Bc* 172560W, *Bc* 95/8201, *Bc* F65185, *Bc* AH1134, *Bc* G9241, *Bt* IBL 200	*Ba* A0248	96.8% (30/31)
F	*Bc* AH603, *Bc* MM3, *Bc* m1293, *Bc* NVH0597-99, *Bc* m1550	–	100% (5/5)
FP	*Bc* ATCC 10,987, *Bc* AH187, *Bc* W, *B. cytotoxicus* NVH 391-98	*Bc* H3081.97, *Bc* F837/76, *Bc* NC7401	57.1% (4/7)
E	*Bc* AH1271, *Bc* Rock1-3, *Bc* Rock3-28, *Bc* Rock3-29, *Bc* ATCC10876, *Bc* ATCC14579, *Bc* E33L, *Bc* Rock3-42, *Bc* Rock4-2, *Bc* AH621, *Bc* Rock1-15, *Bc* AH676, *Bt* Y*BT*-020, *Bt* Al Hakam, *Bt* BGSC 4BD1, *Bt* CT-43, *Bt* 97-27, *Bt* IBL 4222, *Bt* ATCC10792, *Bt* T03a001, *Bt* T13001, *Bt* BGSC 4BA1, *Bt* BGSC 4CC1, *Bt* T01001, *Bt* BGSC 4Y1, *Bt* ATCC35646, *Bt* BMB171, *Bt Bt*407, *Bp* DSM 12442, *Bm* DSM 2048, *Bm* Rock1-4, *Bm* Rock3-17	*Bc* Rock3-44, *Bc* Rock4-18	94.1% (32/34)
U	*Ba* CDC 684, *Ba* Tsiankovskii-I, *Bc* Q1, *Bc* CI, *Bc* BDRD-ST196, *Bc* BDRD-ST24, *Bc* BDRD-ST26, *Bc* ATCC4342, *Bc* BDRD-Cer4, *Bc* BGSC 6E1, *Bt* T04001, *Bt* BGSC 4AJ1, *Bt* BGSC 4AW1, *Bw* KBAB4	*Bc* SJ1	93.3% (14/15)

*E* environment, *F* food, *FP* food poisoning, *C* clinical isolations, *U* unknown source

**Table 2:** Origin, typing and BURST-grouping data of *B. cereus s.l*. strains

Group	Strain	Country	Type of sample	ST	glp	gmk	ilv	pta	pur	pyc	tpi
1	*B.cereus_ATCC4342*	USA	F	38	24	12	50	21	23	31	19
	*B.anthracis_A0248*	Unknown	C	1	1	1	1	1	1	1	1
	*B.cereus_03BB87*	USA	C	78	24	22	33	37	34	38	5
	*B.cereus_03BB108*	USA	U	62	38	1	32	1	18	33	24
	*B.thuringiensis_97-27*	Sarajevo	C	113	62	1	57	52	55	37	43
	*B.cereus_03BB102*	USA	C	11	34	1	32	1	33	37	24
	*B.cereus_g9241*	Unknown	C	78	24	22	33	37	34	38	5
	*B.cereus_CI*	Ivory Coast	E	935	34	1	83	1	18	29	5
	*B.anthracis_Cvac02*	China	U	1	1	1	1	1	1	1	1
	*B.anthracis_PAK-1*	Pakistan	U	1	1	1	1	1	1	1	1
	*B.anthracis_Vollum*	Unknown	U	1	1	1	1	1	1	1	1
	*B.anthracis_2000031021*	USA	U	933	65	1	1	1	1	1	1
	*B.anthracis_HYU01*	South Korea	U	3	2	1	1	1	1	1	1
	*B.anthracis_SVA11*	Unknown	C	3	2	1	1	1	1	1	1
	*B.anthracis_A16*	Unknown	C	1	1	1	1	1	1	1	1
	*B.anthracis_H9401*	South Korea	C	1	1	1	1	1	1	1	1
	*B.anthracis_Sterne chromosome*	Unknown	U	1	1	1	1	1	1	1	1
	*B.anthracis_RA3*	France	U	3	2	1	1	1	1	1	1
	*B.anthracis_V770-NP-1R*	USA	U	2	1	1	2	1	1	2	1
	*B.anthracis_BA1035*	South Africa	U	3	2	1	1	1	1	1	1
	*B.anthracis_BA1015*	USA	U	2	1	1	2	1	1	2	1
	*B.anthracis_Sterne*	Unknown	E	1	1	1	1	1	1	1	1
	*B.anthracis_Pasteur*	Unknown	U	1	1	1	1	1	1	1	1
	*B.anthracis_SK-102*	USA	U	1	1	1	1	1	1	1	1
	*B.anthracis_Ohio ACB*	USA	U	1	1	1	1	1	1	1	1
	*B.anthracis_K3*	Unknown	U	1	1	1	1	1	1	1	1
	*B.anthracis_Vollum 1B*	USA	U	1	1	1	1	1	1	1	1
	*B.anthracis_CDC 684*	Unknown	C	1	1	1	1	1	1	1	1
	*B.anthracis_2002013094*	USA	U	933	65	1	1	1	1	1	1
	*B.anthracis_Canadian_bison*	Canada	U	1	1	1	1	1	1	1	1
	*B.anthracis_Turkey32*	Unknown	C	1	1	1	1	1	1	1	1
	*B.thuringiensis_HD571*	Unknown	U	109	34	1	32	1	51	37	24
	*B.cereus_3a*	Unknown	FP	145	65	1	52	1	1	37	24
	*B.cereus_S2-8*	USA	E	145	65	1	52	1	1	37	24
	*B.cereus_AH820*	Unknown	C	460	65	1	56	1	1	53	24
	*B.cereus_F837/76*	Unknown	C	75	44	1	32	1	18	33	24
	*B.cereus_FT9*	Brazil	E	1262	24	12	50	21	23	58	19
	*B.cereus_D17*	Unknown	F	1263	34	1	124	16	18	33	89
	*B.anthracis_Ames A0462*	USA	U	1	1	1	1	1	1	1	1
	*B.anthracis_Ames Ancestor*	Unknown	E	1	1	1	1	1	1	1	1
	*B.anthracis_Ames chromosome*	Unknown	U	1	1	1	1	1	1	1	1
	*B.anthracis_Ames_BA1004*	USA	U	1	1	1	1	1	1	1	1
2	*B.mycoides_ATCC6462*	Unknown	E	116	25	10	22	53	57	23	44
	*B.thuringiensis_HD-1*	USA	E	10	15	6	10	8	3	7	14
	*B.cereus_NC7401*	Japan	FP	26	3	2	31	5	16	3	4
	*B.cytotoxicus_391-98*	Unknown	FP	930	211	127	221	221	57	10	171
	*B.weihenstephanensis_KBAB4*	Unknown	E	958	18	10	79	36	77	70	18
	*B.weihenstephanensis_WSBC10204*	Germany	F	196	64	10	79	36	56	22	11
	*B.thuringiensis_HD1002*	Israel	U	16	15	7	7	2	6	8	13
	*B.thuringiensis_Bt407*	Unknown	U	10	15	6	10	8	3	7	14
	*B.thuringiensis_IS5056*	Unknown	E	10	15	6	10	8	3	7	14
	*B.thuringiensis_CT-43*	Unknown	U	10	15	6	10	8	3	7	14
	*B.thuringiensis_HD-771*	Unknown	U	12	15	7	7	2	7	10	13
	*B.thuringiensis_HD-789*	Unknown	U	16	15	7	7	2	6	8	13
	*B.cereus_AH187*	Unknown	FP	26	3	2	31	5	16	3	4
	*B.cereus_G9842*	Unknown	C	56	15	7	7	2	7	26	13
	*B.thuringiensis_YBT-1518*	China	E	1261	15	143	10	242	4	7	14
3	*B.cereus_ATCC14579*	USA	FP	921	13	125	8	11	11	12	169
	*B.thuringiensis_HD-29*	Czechoslovakia	U	15	9	8	16	13	2	16	9
	*B.thuringiensis_HD73*	Unknown	U	8	7	8	16	13	2	16	7
	*B.thuringiensis_YBT-1520*	Unknown	E	8	7	8	16	13	2	16	7
	*B.thuringiensis_BMB171*	Unknown	U	184	12	8	8	14	9	12	7
	*B.thuringiensis_Wang*	Unknown	E	18	11	9	14	12	12	14	7
	*B.cereus_FORC-005*	South Korea	FP	998	33	8	13	11	8	17	7
	*B.cereus_B4264*	Unknown	C	89	14	8	40	19	2	17	17
4	*B.cereus_ATCC10987*	Canada	F	32	5	4	3	4	15	6	16
	*B.cereus_NC7401*	Japan	FP	26	3	2	31	5	16	3	4
	*B.cereus_AH187*	Unknown	FP	26	3	2	31	5	16	3	4
	*B.cereus_FRI-35*	Unknown	U	90	6	4	41	5	43	46	3
	*B.cereus_Q1*	Unknown	E	266	3	2	21	17	36	3	4
5	*B.toyonensis_BCT-7112*	Japan	U	111	43	26	35	42	39	41	30
	*B.thuringiensis_MC28*	Unknown	E	158	72	42	69	42	63	41	30
6	*B.pseudomycoides_DSM12442*	Unknown	U	83	63	13	58	23	25	44	35
	*B.cereus_Al Hakam*	Iraq	E	260	106	59	58	88	99	78	66
7	*B.cereus_E33L*	Namibia	E	908	115	124	119	116	119	108	88

Detailed allelic profiles for the seven housekeeping genes (*glp, gmk, ilv, pta, pur, pyc, tpi*) are given for the ST (Sequence Type). The sequences of strains are from NCBI databases. Numbers were arbitrary assigned to allele fragment for each locus. The STs were grouped by BURST analysis: 75 strains were divided into seven groups based on the number of differences in the allelic profiles (Table 1)

Abbreviations are as follows: *C* clinical isolates, *E* environmental isolates, *F* food isolates, *FP* food poisoning isolates, *U* strains with unknown origin

*ST* stands for Sequence Type and corresponds to the specific allelic profile

### MLST analysis

Seven loci encoding housekeeping genes were chosen for MLST analysis: *glp* (glycerol kinase), *gmk* (guanylate kinase), *ilv* (isoleucine-valine), *pta* (phosphate acetyltransferase), *pur* (purine synthesis), *pyc* (pyruvate carboxylase), *tpi* (triosephosphate isomerase) (Lampe and English 2016). 75 strains for which the *nheABC* genes are available in the genome databases, were selected for the MLST analysis (Table 2). The MLST result were shown in minimum-spanning tree (http://pubmlst.org/analysysis/). The nucleotide sequence diversity of *glp, gmk, ilv, pta, pur, pyc, tpi* and *nheABC* genes was analyzed at two levels: by constructing a phylogenetic tree for each loci with the CLC Main Workbench 7 software (ClCbio, a Qiagen Company) using the neighbor joining (NJ) algorithm with Jukes Cantor as substitution rate model, and by building sequence types (ST) of the various strains using the non-redundant database (NRDB) for allele comparison (http://pubmlst.org/). To cluster the strains according to their ST, BURST analysis was performed which defines a group when at least 5/7 loci were identical (http://pubmlst.org/perl/mlstanalyse/). Sequences of the seven chromosomal loci were also concatenated with or without adding the *nheABC* genes to construct and compare the respective phylogeny trees generated by CLC Main Workbench 7 software using UPGMA algorithm and Kimura 80 mathematical model using. The correctness of the results was evaluated using a 100-step bootstrap test (Virginie et al. 2015).

## Results and discussion

### *nheABC* occurrence among the *B. cereus s.l*. strains

While the mechanisms involved in the *B. cereus* diarrhoeal pathogenesis are still largely unknown, a large panel of enterotoxins have been designated as potential causative agents led to this syndrome. And NHE is regularly cited as a candidate because of its cytotoxic, necrotic and haemolytic activities on human intestinal cell lines (Lindback et al. 2004; Zhu et al. 2016). Some *B. cereus* genomes harbor the nhe operon which codes for the cytolytic protein NheA and the binding components NheB and NheC (Wehrle et al. 2009). In this study, the *nheABC* sequence from *B. cereus* ATCC14579 were applied to find all the homologous genes among the 174 strains by BLAST program in http://blast.ncbi.nlm.nih.gov/Blast.cgi. Since the first bacterial genome sequence was completed in 1995, 174 sequences of *Bacillus* strains genomes have been published in NCBI database until Aug. 2015 (the number of assembled and annotated *B. cereus* genome is 294 until Dec. 2016). The *nheABC* loci were found in 81 strains, including all the 31 *B. anthracis* strains, 24 *B. cereus* strains, 21 *B. thuringiensis* strains, 2 *B. weihenstephanensis* strains, 1 *Bacillus bombysepticus* strain and 1 *Bacillus toyonensis* strain. 2 *B. mycoides* strains were sequenced but only one strain contains *nheABC* loci.

However, *Bacillus amyloliquefaciens, Bacillus atrophaeus, Bacillus clausii, Bacillus coagulans, Bacillus infantis, Bacillus lehensis, Bacillus licheniformis, Bacillus megaterium, Bacillus methanolicus, Bacillus methylotrophicus, Bacillus pseudofirmus, Bacillus pumilus, Bacillus sp., Bacillus subtilis, Bacillus halodurans, Geobacillus kaustophilus* and *Bacillus cellulosilyticus* do not contain *nheABC* loci (Table 1). The *nheABC* operon occurrence in NCBI database is 46.6% (81/174). And the mean value frequency of *nheA, nheB, nheC* found in the literature is 82, 81 and 78%, respectively (Swiecicka et al. 2006; Gaviria et al. 2000; Hansen and Hendriksen 2001; Banerjee et al. 2011; Moravek et al. 2004; De Jonghe et al. 2010; Krause et al. 2010; Zhou et al. 2010; Samapundo et al. 2011; Chon et al. 2015). These frequencies are slightly higher than the ratio we measured from database. Maybe because more and more *Bacillus* genomes sequencing are completed, and *nheABC* operon is absent from the recently released genomes, so the ratio measured from database is low.

### Genomic diversity of *nheABC*

81 genetic regions (30 kb in size) from the *nheABC* ^+^ strains mentioned above centered on the *nheABC* operon were collected and aligned for genomic diversity analysis. The putative ORFs (more than 200aa) from all fragments were annotated (Table 3), and compared to illustrate their genetic features. By using the *nheABC* locus of *B. cereus* ATCC14579 as a typical reference, good conservation was observed in the upstream region of this gene cluster among the other 76 strains. Interestingly, the rest four strains showed different pattern of conservation, therefore, we studied the genomic diversity in two parts.

**Table 3:** Annotation of genes around *nheABC* genomic loci

Identifier	Gene function	Identifier	Gene function
247	Two-component response regulator vanR	551	multicopper oxidase family protein
226	sensor histidine kinase, C terminus	216	CAAX amino protease
262	M24/M37 family peptidase	235	No published record
431	manganese transport protein MntH	291	No published record
306	hypothetical protein BA_1881	316	inosine/uridine-preferring nucleoside hydrolase
491	No published record	318	Virginiamycin B lyase
314	2-dehydropantoate 2-reductase	591	S-layer domain protein
262	conserved hypothetical protein	439	Aminopeptidase
218	amino acid permease	456	carboxylic ester hydrolase
334	non-hemolytic enterotoxin A	206	Transcriptional regulator, TetR
402	non-hemolytic enterotoxin B	207	acetyltransferase
305	non-hemolytic enterotoxin C	209	CAAX amino protease
208	homoserine/threonine efflux protein	364	No published record
315	deoxyribonucleoside regulator DeoR	211	No published record
223	deoxyribose-phosphate aldolase	569	DNA topoisomerase III
393	nucleoside transporter NupC	1152	molybdate metabolism regulator
437	pyrimidine-nucleoside phosphorylase	368	No published record
316	membrane protein, putative	768	No published record
226	No published record	368	VWA domain containing CoxE-like protein
294	BNR repeat-containing protein	524	zinc finger, swim domain protein
427	xaa-pro aminopeptidase		

**Fig. 1:**
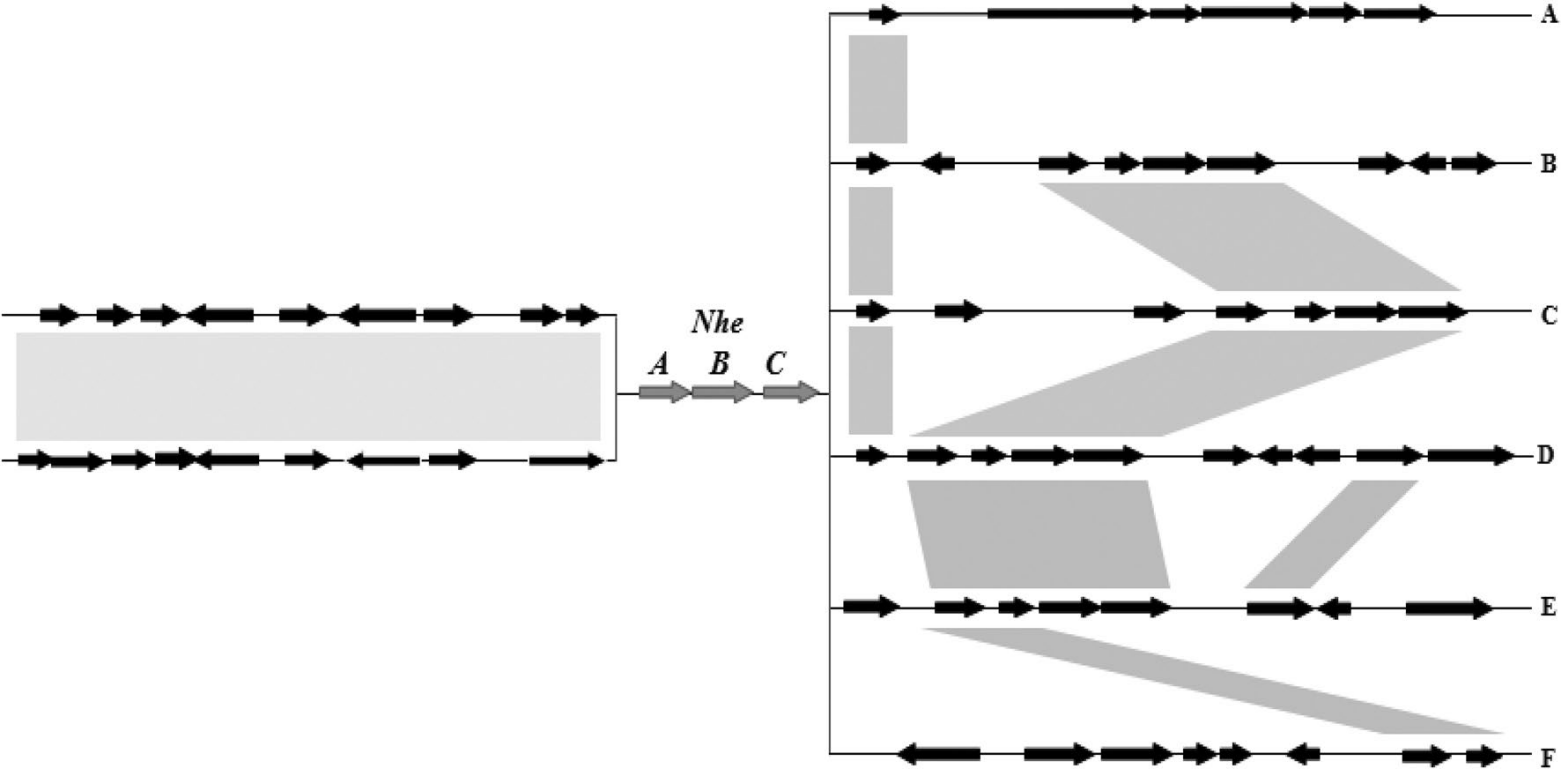
Genomic diversity of the 30 kb fragments centered on *nheABC* locus for forty-five *B. cereus s.l*. group strains (including 10 *Ba*, 22 *Bc*, 11 *Bt*, 1 *Bm* and 1 *Bw*). The alignment of the available sequences in database revealed a much higher degree of conservation in the upstream region in terms of gene content, compared to the downstream region. Orthologous genes are indicated by *arrows* of the same size and matches are indicated in *gray shadow*

In the first part, 30 kb DNA sequences centered on the *nheABC* loci from 76 strains were aligned and labelled in the sketch map. The alignment result showed a higher degree of conservation in the upstream region in terms of gene content, relative to the downstream region (Fig. 1). The downstream of *nheABC* locus contained six distinct conserved regions, which was different from their gene contents and organizations (indicated as A to F, and their relative frequencies of 5/76, 24/76, 2/76, 12/76, 31/76 and 2/76, respectively). Specifically, Branch A contained *B. thuringiensis* YBT-1520, HD-29, HD-1, HD-73 and *B. mycoides* ATCC6462. Branch B contained *B. cereus* AH820, 03BB102, AH187, ATCC10987, etc. strains. Branch C contained *B. cereus* NC7401 and Q1. Branch D contained *B. cereus* ATCC14579, AH187, 03BB102, etc. strains. Branch E contained all the 31 *B. anthracis* strains. Branch F contained *B. weihenstephanensis* KBAB4 and WSBC10204.

In the *nheABC* locus upstream regions (left part of *nheABC* loci in Fig. 1), seven genes (two-component response regulator vanR, C terminus of sensor histidine kinase, M24/M37 family peptidase, manganase transport protein MntH, sulfur transferase, hypothetical protein and 2-dehydropantoate 2-reductase) were present in all the 76 fragments. Interestingly, in 49 of the 76 strains, a 505aa ORF (amino acid permease) was found immediately upstream of the NheA, while it was broken up into two smaller ORFs (also annotated as amino acid permease) in the 32 remaining strains, including which included 31 *B. anthracis* strains plus *B. thuringiensis* 97-27 strain.

In the *nheABC* locus downstream regions (right part of *nheABC* loci in Fig. 1), there was obvious genes constituent change and rearrangement in different branch. The gene *rhtB* (homoserine/threonine efflux protein) was conserved in Branch A–D, but disappeared in Branch E and F. The four genes cluster *deoR* (deoxyribonucleoside regulator), *deoC* (deoxyribose-phosphate aldolase), *nupC* (nucleoside transporter), *pdp* (pyrimidine-nucleoside phosphorylase) was well kept in the same direction in Branch B–F. And the gene *yndJ* encoded a putative membrane protein also maintained in Branch D and E. The other genes showed different variety. In the downstream regions, the composition and arrangement of genes diversified in different Branch strains.

But the rest five *nheABC* ^+^ strains, *B. cereus* ATCC4342, D17, FT9, G9241 and *B. cytotoxicus* NVH391-98, sequences showed high genomic diversity, could not be included into any branch.

**Fig. 2:**
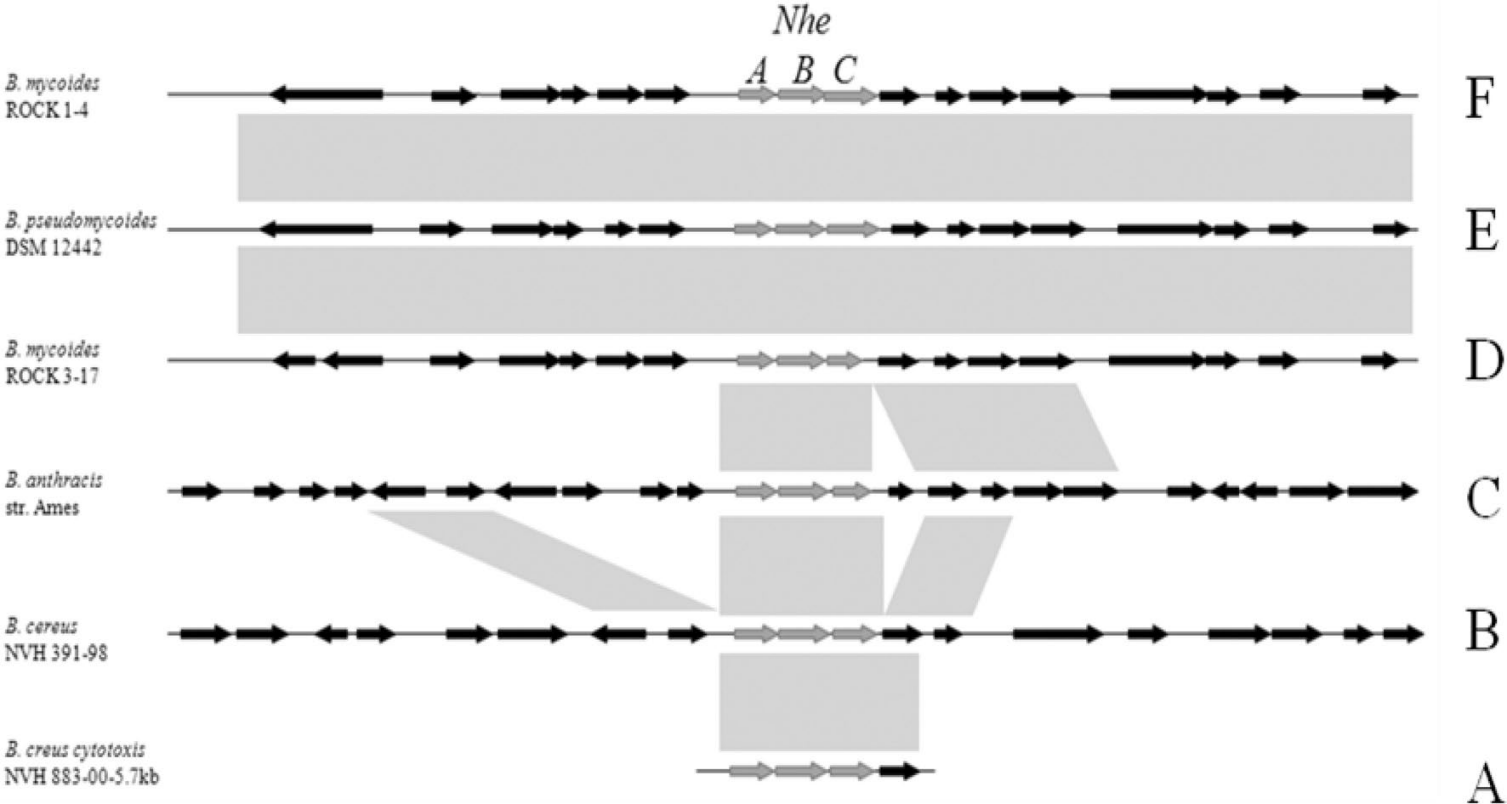
Comparison of the 30 kb fragment surrounding the *nheABC* locus from *B. anthracis* Ames with the corresponding *nheABC* regions from *B. mycoides* Rock1-4, 3-17, *B. pseudomycoides* DSM12442, *B. cereus* NVH391-98 and a 5.7 kb fragment from *B. cereus* NVH883-00. Orthologous genes are indicated by *arrows* of the same size and matches are indicated in *gray*

To illustrate genes distribution of the whole 30 kb fragments in different branch, six representative strains were selected. Based on the 5.7 kb fragment from *B. cereus* NVH883-00, genes arrangement surrounding the *nheABC* locus from *B. anthracis* Ames with the counterparts from *B. mycoides* Rock1-4, *B. pseudomycoides* DSM12442, *B. mycoides* Rock 3-17, *B. cereus* NVH391-98 were shown in Fig. 2. All orthologous genes are indicated by arrows of the same size and matches are indicated in gray in the sketch map. *B. mycoides* Rock1-4, *B. pseudomycoides* DSM12442 and *B. mycoides* Rock 3-17 had almost the same genes distribution. It is easy to recognize gene cluster rearrangement and gene insertion in *B. anthracis* Ames and *B. cereus* NVH391-98, though these two strains shared high similarity in most genes rearrangement.

### Genetic diversity of NheA, B and C

To further explore the genetic diversity based on the 81 collected NheA, B and C protein sequences, each protein was analyzed, respectively. The size of 73 NheA proteins was 386aa, and the size of the rest eight strains, such as *B. cytotoxicus* NVH391-98, *B. cereus* G9241, FT9, Ba HYU01, SVA11, RA3, *B. anthracis* BA1035, 2002013094 was 387aa, 312aa, 279aa, 388aa, 388aa, 388aa, 388aa and 318aa, respectively. Based on sequences diversity, all the 73 NheA proteins were divided into four groups (Fig. S1). In group A, the NheA from different strains shared 97–100% identities with *B. cereus* ATCC14579-NheA. In group B, the NheA from strains *B. cereus* G9241, *Bw* WSBC10204, KBAB4, *Ba* 2002013094, *B. toyonensis* BCT-7112 and *Bm* ATCC6462 shared 97, 96, 97, 97, 96 and 95% identities with *B. cereus* ATCC14579-NheA, respectively. In group C, the 312aa NheA from strain *B. cereus* G9241 shared 97% identity with *B. cereus* ATCC14579-NheA. Moreover, the coverage of *B. cereus* G9241 and *Ba* 2002013094 was only 78 or 82%, respectively. In group D, the 387aa NheA proteins from *B. cereus* NVH391-98 shared 78% identity with *B. cereus* ATCC14579-NheA.

NheB genetic diversity seemed more conservative. The size of 79 NheB proteins was 402aa, and the size of the rest two strains (*B. cytotoxicus* NVH391-98, *B. cereus* CI) was 401aa. Based on sequences diversity, all the 81 NheB sequences were divided into three groups (Fig. S2). In group A, the NheB from different strains shared 99–100% identities with *B. cereus* ATCC14579-NheB. In group B, the NheB from strains *B. mycoides* ATCC6462 shared 98% identity with *B. cereus* ATCC14579-NheB. In group C, the 401aa NheB proteins from *B. cereus* NVH391-98 shared 87% identity with *B. cereus* ATCC14579-NheB.

NheC genetic diversity analysis showed much less conservation, compared to NheA and NheB. The NheC size from the 81 strains ranged from 305aa to 397aa. Most of the proteins (74 out of 81) were 359aa. The size of the rest seven strains, such as *Ba* Vollum, CDC684, Han, *B. cytotoxicus* NVH391-98, *B. cereus* 4342, FT9 and *B. thuringiensis* AI Hakam was 305aa, 305aa, 353aa, 353aa, 362aa, 362aa, 397aa and 362aa, respectively. Based on sequences diversity, all the 81 NheC were divided into three groups (Fig. S3). In group A, the NheC from different strains shared 94–100% identities with *B. cereus* ATCC14579-NheC. In group B, the NheC proteins from *Bw* WSBC10204 and KBAB4 both shared 92% identity with *B. cereus* ATCC14579-NheC. In group C, the NheC from *Ba* Han shared 86% identity with *B. cereus* ATCC14579-NheC. In group D, the NheC protein from *B. cereus* NVH391-98 shared 73% identity with *B. cereus* ATCC14579-NheC.

In all the analysis of NheA, B and C genetic diversity, the strain *B. cereus* NVH391-98 was always exclusively different from other strains. This is in agreement with the observations performed on the genomic diversity of *nheABC* loci (Böhm et al. 2015).

### Seven housekeeping genes MLST analysis from 75 *nheABC*^+^ strains

To further characterize the sequence variation among the 75 *nheABC* positive strains of *B. cereus*, MLST was performed using the seven housekeeping genes: *glp, gmk, ilv, pta, pur, pyc* and *tpi*. The sequence variability of each locus was also studied in details. Based on the allelic profiles of the seven loci, ST could be defined for all the isolates. The high number of ST is another illustration of the high diversity existing among the *nheABC* positive *B. cereus s.l*. strains. Of note, Table S1 displays the correspondence between the allelic profiles defined in this work and those reported in previous MLST schemes (Helgason et al. 2004; Sorokin et al. 2006). The 75 isolates (Table S1) were then subjected to a BURST analysis to group the strains according to the similarity of their allelic profile. The isolates were grouped together when five out of the seven analyzed loci were identical. Based on this criterion, seven clusters were formed, as shown in Table 2. 75 strains were clustered in seven groups. Strains coming from foodborne outbreaks spread in different groups. This result showed that there was no obvious association between similar allelic profiles and geographic or source origins.

**Fig. 3:**
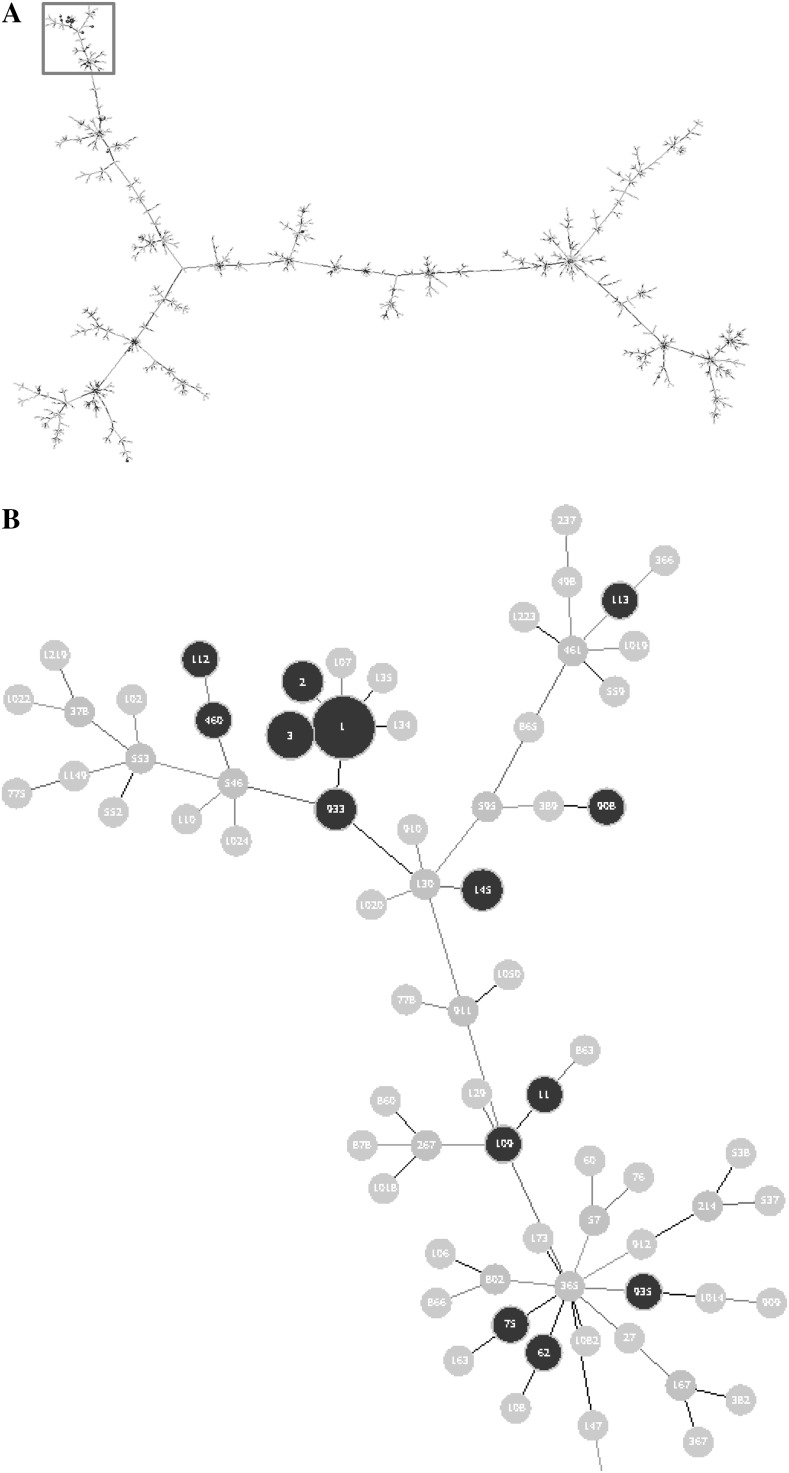
MLST split tree of *Bacillus cereus*. The location of each strain illustrated the number of locus differences with other strains in MLST split tree. The distances represented the number of locus differences between every pair of samples. The *size of the circles* indicated the observed frequencies. The *black circles* represented the strains from Table S1, the *blue* and *green circles* were 2033 *Bacillus cereus* strains (data not shown). The *numbers in the circles* were ST of each strains. **a** the whole tree. **b** the partial enlargement of A

To visualize the influence of *nheABC* gene on the relatedness of the strains, 75 strains displaying *nheABC* positive (originating from different countries) were selected for the MLST analysis of the *Bacillus* strains. A tree was built with the sequences of the seven loci concatenated with *nheABC*. PHYLOViZ was built upon the goeBURST implementation (available at http://goeburst.phyloviz.net) and it allows to integrate and display multiple sources of information. The result was showed in Fig. 3. The whole tree was built on all the selected species about 2033 *Bacillus cereus* strains from different sources (data not shown), and the 75 selected strains assigned black color were located in this split tree. Though the observed frequency of each strain was different, all the selected strains spread randomly in all the part of MLST split tree. The number of locus differences between each pair strains could be observed from the line distances of the tree. This explained that the *nheABC* sequences variation had no favor of any type of strains, and also showed again that there was no obvious association between similar allelic profiles and geographic or source origins.

To verify that *nheABC* operon was not prone to lateral transfer, 30 kb genome fragments centered on the *nheABC* operon locus were investigated. Although the genomic neighborhood of *nheABC* showed some variability, no element potentially involved in horizontal transfer was found (data not shown). The comparison of the 76 *Bacillus* fragments has provided valuable insights into defining the key genetic complement of the organisms, which forms the basic genetic support to define the organism’s pathogenicity ability (Lisdawati et al. 2015).

**Fig. 4:**
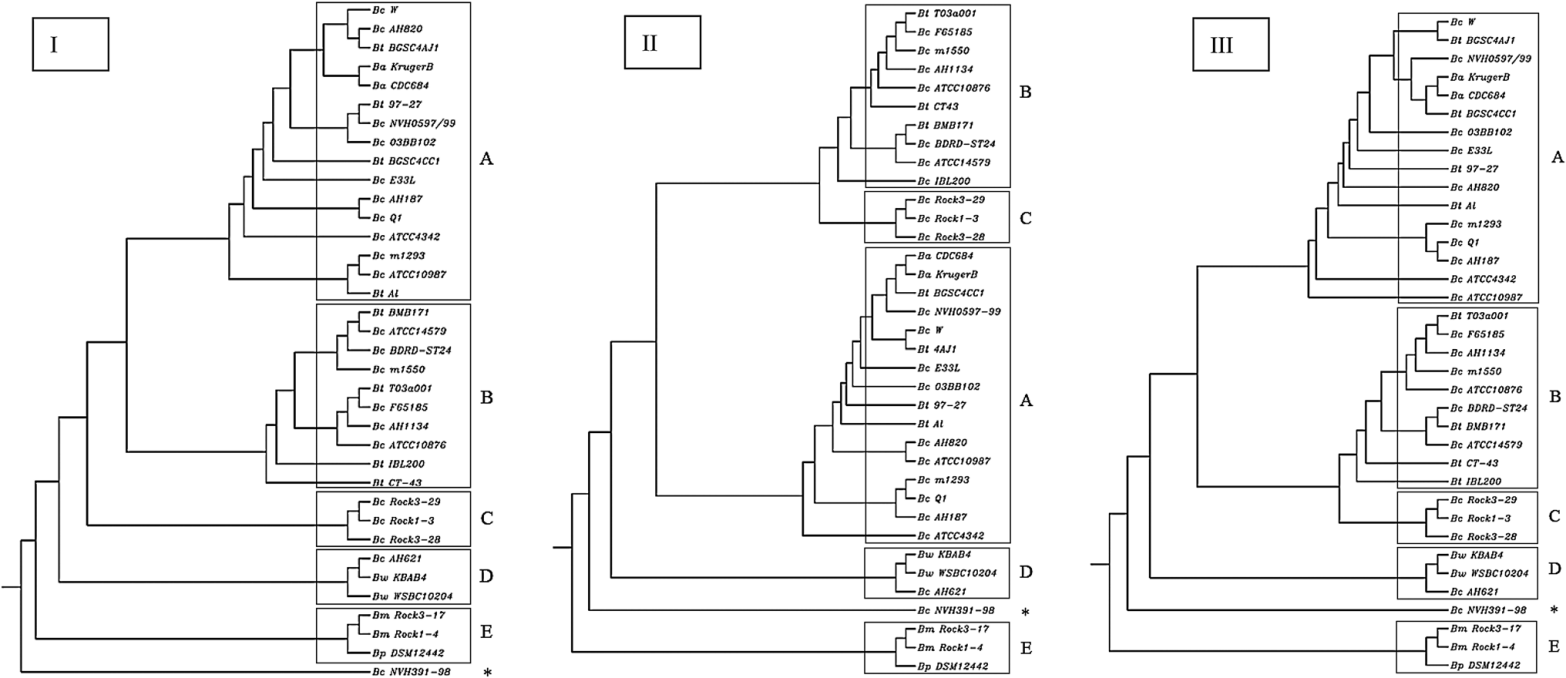
The NJ trees built based on the concatenated sequences of the six housekeeping genes and *nheABC* genes. I (based on six housekeeping genes), II (based on *nheABC* genes) and III (based on the concatenation of six housekeeping genes and *nheABC* genes)

To investigate the genetic diversity of *nheABC* and seven housekeeping genes (*glp, gmk, ilv, pta, pur, pyc* and *tpi*), the concatenated sequences of the seven housekeeping genes (I), *nheA, B* and *C* genes (II), the concatenated sequences of the seven housekeeping genes plus the *nheA, B* and *C* genes (III) were collected from 35 *B. cereus s.l*. group strains for which the seven genes sequences were all available in NCBI database. The sequences were then analyzed by online software clustalw (http://www.genome.jp/tools/clustalw/). The neighbor joining trees based on seven housekeeping genes together with *nheA, B* and *C* genes were built. In tree I (Fig. 4), *B. cereus* NVH391-98 was far away from all the other 35 strains. Genes from different strains clustered together, according to the genetic relationship five main clusters are noted as A–E. In tree II and III, though the order of five main clusters changed, the constituent of each cluster stayed the same. The structure of these trees were similiar to published *Bacillus* phylogenetic trees (Virginie et al. 2015). This result indicated that no striking differences was observed between the various trees, and meant that the influence of *nheABC* on strain clustering is limited. This result also showed that the genetic determinants of the NHE had no any obvious relationship with the *nheABC* genes sequence of a strain and its virulence in the diarrhoeal pathogenesis.

In this study, we found that the genetic determinants of the NHE toxin did not bring any obvious link between the *nheABC* genes sequence of a strain and its virulence in the diarrhoeal pathogenesis. To assess whether NHE is a significant factor in this disease, a transcriptomic study should be considered to take the genes expression of the toxin into account. And the potential action of NHE also should be investigated in concert with other possible enterotoxins (e.g., HBL, CytK or HlyII) and other virulence factors to evaluate the diarrhoeic potential of *B. cereus* strains. To elucidate the actual involvement of these molecules in the diarrhoeal syndrome, it is necessary to find an adequate animal model. In fact, due to their proteinaceous nature, these putative enterotoxins may be prone to a rapid inactivation in the intestinal tract, unless they would be released by the *B. cereus* cells in the immediate vicinity of host’s intestinal epithelium being protected by the mucus layer. However, this hypothesis has still to be verified.

## Conclusions

The *nheABC* genes do not affect the diversity displayed by housekeeping genes, and this specific protein is probably not implicated in the diarrheal syndrome. Our data provide a scientific basis for us to know more about *nheABC* loci in food poisoning *B. cereus*.

## Electronic supplementary material

Below is the link to the electronic supplementary material.

Supplementary material 1 (DOC 147 KB)
