# Supplementary material for: Genetic and genomic diversity of NheABC locus from Bacillus strains
Source: Arch Microbiol. 2017 Mar 10;199(5):775–85. doi: 10.1007/s00203-017-1350-9 (PMC5486940; doi:10.1007/s00203-017-1350-9)
Supplement: Supplementary file 1 — Supplementary material 1 (DOC 147 KB) [file 203_2017_1350_MOESM1_ESM.doc]

Table S1 Information of the strains selected for MSLT analysis.

| ID | Isolate | Variety serovar | Country | Year | Source | ST | Type of  sample |
| --- | --- | --- | --- | --- | --- | --- | --- |
| 16 | ATCC 10987 | cereus | Canada | 1930 | milk products | 32 | F |
| 22 | ATCC 4342 | cereus | USA | 1900 |  | 38 | F |
| 23 | ATCC 14579 | cereus | USA | 1916 |  | 4 | FP |
| 111 | ames | anthracis |  |  |  | 1 | C |
| 124 | 03BB87 | cereus | USA | 2003 | blood | 78 | C |
| 140 | 03BB108 | cereus | USA | 2003 | Environment | 62 | U |
| 190 | ATCC 6462 | mycoides |  |  |  | 116 | E |
| 260 | 03BB102 | cereus | USA | 2003 | blood | 11 | C |
| 544 | NC7401 | cereus | Japan | 1974 | cereulide producer | 26 | FP |
| 656 | g9241 |  |  |  |  | 78 | C |
| 1581 | E33L | cereus | Namibia | 1996 | a dead zebra carcass | 908 | E |
| 1627 | ATCC 14579 | cereus | USA | 1916 |  | 921 | FP |
| 1634 | KBAB4 | weihenstephanensis | Germany | 1998 |  | 958 | E |
| 1914 | WSBC 10204 | weihenstephanensis | China | 2013 | Milk | 196 | F |
| 1915 | Cvac02 | anthracis | Pakistan | 1978 | blood | 1 | U |
| 1916 | PAK-1 | anthracis |  |  | sheep | 1 | U |
| 1917 | Vollum | anthracis | USA |  |  | 1 | U |
| 1918 | 2000031021 | anthracis | South Korea | 2009 | soil | 933 | U |
| 1919 | HYU01 | anthracis |  |  | soil | 3 | U |
| 1920 | SVA11 | anthracis |  |  |  | 3 | C |
| 1921 | A16 | anthracis | South Korea |  |  | 1 | C |
| 1922 | H9401 | anthracis |  |  | cutaneous anthrax patient | 1 | C |
| 1923 | Sterne | anthracis | France | 1998 |  | 1 | E |
| 1924 | RA3 | anthracis | USA | 1951 | Bovine | 3 | U |
| 1925 | V770-NP-1R | anthracis | South Africa |  | Bovine | 2 | U |
| 1926 | BA1035 | anthracis | USA | 1939 | Human | 3 | U |
| 1927 | BA1015 | anthracis |  |  | Bovine | 2 | U |
| 1928 | Sterne | anthracis |  |  |  | 1 | E |
| 1929 | Pasteur | anthracis | USA | 1976 | Cat | 1 | U |
| 1930 | SK-102 | anthracis | USA |  | Wool from Pakistan | 1 | U |
| 1931 | Ohio ACB | anthracis |  |  | Pig | 1 | U |
| 1932 | K3 | anthracis | USA | 1951 | Human | 1 | U |
| 1933 | Vollum 1B | anthracis |  |  | Human | 1 | U |
| 1934 | CDC 684 | anthracis |  |  |  | 1 | C |
| 1935 | A0248 | anthracis | USA | 1956 |  | 1 | C |
| 1936 | 2002013094 | anthracis | Canada |  | Soil | 933 | U |
| 1937 | Canadian_bison | anthracis |  |  | Bison | 1 | U |
| 1938 | Turkey32 | anthracis | Japan | 1966 |  | 1 | C |
| 1939 | BCT-7112 | toyonensis |  | 2006 | soil | 111 | U |
| 1940 | 97-27 | thuringiensis |  |  | human tissue | 113 | C |
| 1941 | HD571 | thuringiensis | India | 1915 |  | 109 | U |
| 1942 | HD1011 | thuringiensis | Czechoslovakia | 1970 |  | 112 | U |
| 1943 | HD-29 | thuringiensis | Israel |  | Dendrolimus sibericus | 15 | U |
| 1944 | HD1002 | thuringiensis |  | 1987 | Sewage | 16 | U |
| 1945 | BGSC 4AA1 | thuringiensis |  |  | soil | 23 | U |
| 1946 | Bt407 | thuringiensis |  |  |  | 10 | U |
| 1947 | MC28 | thuringiensis |  |  |  | 158 | E |
| 1948 | HD73 | thuringiensis |  |  |  | 8 | U |
| 1949 | IS5056 | thuringiensis |  |  |  | 10 | E |
| 1950 | YBT-1520 | thuringiensis |  |  |  | 8 | E |
| 1951 | BMB171 | thuringiensis |  |  |  | 184 | U |
| 1952 | CT-43 | thuringiensis |  |  |  | 10 | U |
| 1953 | HD-771 | thuringiensis |  |  |  | 12 | U |
| 1954 | HD-789 | thuringiensis |  |  |  | 16 | U |
| 1955 | Wang | bombysepticus |  |  |  | 18 | E |
| 1956 | AH187 | cereus |  |  |  | 26 | FP |
| 1957 | G9842 | cereus |  |  |  | 56 | C |
| 1958 | FRI-35 | cereus |  |  |  | 90 | U |
| 1959 | 3a | cereus | Iraq |  |  | 145 | FP |
| 1960 | Al Hakam | cereus | USA | 1993 |  | 260 | E |
| 1961 | S2-8 | cereus | South Korea | 2014 | soil | 145 | E |
| 1962 | FORC_005 | cereus |  |  | chicken cutlett | 998 | FP |
| 1963 | AH820 | cereus |  |  |  | 460 | C |
| 1964 | Q1 | cereus |  |  |  | 266 | E |
| 1965 | F837/76 | cereus |  |  |  | 75 | C |
| 1966 | CI | anthracis |  | 1969 |  | 935 | E |
| 1967 | B4264 | cereus |  |  |  | 89 | C |

Fig. S1 Phylogenetic tree of NHEA protein from 76 selected the *Bacillus* strains.

Fig. S2 Phylogenetic tree of NHEB protein from 76 selected the *Bacillus* strains.

Fig. S3 Phylogenetic tree of NHEC protein from 76 selected the *Bacillus* strains.
